# The Influence of Time on the Sensitivity of SARS-CoV-2 Serological Testing

**DOI:** 10.21203/rs.3.rs-1286644/v1

**Published:** 2022-02-17

**Authors:** Arturo Torres Ortiz, Fernanda Fenn Torrente, Adam Twigg, James Hatcher, Anja Saso, Tanya Lam, Marina Johnson, Helen Wagstaffe, Rishi Dhillon, Anabelle Lea Mai, David Goldblatt, Rachel Still, Matthew Buckland, Kimberly Gilmour, Louis Grandjean

**Affiliations:** Great Ormond Street Hospital; University College London; University of Cambridge; Great Ormond Street Hospital; London School of Hygiene & Tropical Medicine; Great Ormond Street Hospital; Great Ormond Street Hospital; Great Ormond Street Hospital; University Hospital of Wales; Great Ormond Street Hospital; University College London; Swansea Bay University Health Board; Great Ormond Street Hospital; Great Ormond Street Hospital; Great Ormond Street Hospital

**Keywords:** SARS-CoV-2, COVID-19, virus, nucleoprotein, spike protein, antibody, assay, serology

## Abstract

**Background:**

Serological testing is used to quantify SARS-CoV-2 seroprevalence, guide booster vaccination and select patients for anti-SARS-CoV-2 antibodies therapy. However, our understanding of how serological tests perform as time passes after infection is limited.

**Methods:**

Four assays were compared in parallel: 1) the multiplexed spike, nucleoprotein and receptor binding domain Meso Scale Discovery (MSD) assay 2) the Roche Elecsys-Nucleoprotein assay (Roche-N) 3) the Roche Spike assay (Roche-S) and 4) the Abbott Nucleoprotein assay (Abbott-N) on serial positive monthly samples from hospital staff up to 200 days following infection as part of the Co-Stars study.

**Results:**

We demonstrate that 50% of the Abbott-N assays give a negative result after 175 days (median survival time 95% CI 168–185 days) while the Roche-N assay (93% survival probability at 200 days, 95% CI 88–97%) maintained seropositivity. The MSD spike (97% survival probability at 200 days, 95% CI 95–99%) and the Roche-S assay (95% survival probability at 200 days, 95% CI 93–97%) also remained seropositive. The best performing quantitative Roche-S assay showed no evidence of waning Spike antibody titres over 200-days.

**Conclusions:**

The Abbott-N assay fails to detect SARS-CoV-2 antibodies as time passes since infection. In contrast the Roche and the MSD assays maintained high sensitivity. The limitations of the Abbott assay must be considered in clinical decision making. The long duration of detectable neutralizing spike antibody titres by the quantitative Roche-S assay provides further evidence in support of long-lasting SARS-CoV-2 protection to pre-existing strains of SARS-CoV-2 following natural infection.

## Introduction

Following natural infection or vaccination, sensitive measurement of SARS-CoV-2 serological status is important to identify immune correlates of protection from future waves of the pandemic, evaluate those in need of booster vaccination and identify candidates for SARS-CoV-2 antibody therapy. The rapid response to the COVID-19 pandemic has led to the development of a wide range of serological tests suitable for evaluating SARS-CoV-2 exposure, infection or vaccination status [1–3]. Typically, these tests are approved for use by the regulatory authorities based on their performance against a panel of reference sera including positive and negative controls at either 14- or 21-days post infection [4].

Public Health England reported a 93.9% sensitivity for the Abbott SARS-CoV-2 IgG Nucleoprotein assay [5] and 100% for the Roche Elecsys Nucleoprotein assay at ≥14 days post infection [6]. This led to widespread adoption of these tests across NHS laboratories for testing at population level. Other studies have confirmed this test performance at 14–21 days post infection [7, 8]. Population level serological studies have also based their conclusions - vital to guide national policy - on the basis of these tests [9] without considering how time since infection influences the performance of the test. The problem with this approach is that it does not take into account SARS-CoV-2 humoral dynamics and changes in avidity over time [10, 11]. Although serological tests with limited diagnostic range may demonstrate excellent sensitivity shortly after infection, it is unclear how they will perform with time following infection or vaccination.

In order to address this question, we applied 4 widely used serological assays in parallel to serial samples from the Co-STARs study [12] in which staff testing seropositive to SARS-CoV-2 were followed for up to 200 days following infection. We compared the proportion of samples that remained seropositive over time using a survival analysis and determined the decay rate of the nucleoprotein (N) antibody and the spike (S) antibody for each test using a previously published mathematical model fitted to the data.

## Materials And Methods

### Study setting and design

Serological testing was performed on stored serum samples collected as part of the Co-STARs study (www.clinicaltrials.gov, NCT04380896) run at Great Ormond Street Hospital between April and November 2020 [10]. The study had national Integrated Research Application System (IRAS) approval and all experimental protocols were approved. Briefly, Co-STARs was a 1-year single-centre prospective cohort study of antibody responses to COVID-19 infection in healthcare workers. Serum samples were taken from the 3657 participants at baseline and underwent a screening ELISA using the EDI assay. Repeated monthly serum samples were then taken from those with a seropositive baseline screening test for up to 250 days after the date of infection. Those samples identified as seropositive with available symptom start date had further confirmatory testing with the quantitative three antigen MSD assay.

### Study participants:

The majority of hospital staff were eligible for the Co-STARS study [12]. Only those participants with significant immunosuppression, those that had received blood products within 6 months of recruitment and those that had active and ongoing symptoms of SARS-CoV-2 infection (within the last 21 days) were excluded. Only samples from individuals with at least one positive test from any platform were included in the analysis. Moreover, individuals without a known symptom start date were removed.

### Data Collection

As part of the Co-STARs study all participants undertook a detailed standardised online questionnaire at study entry [12]. This included the date of onset of COVID-19 symptoms, and any SARS-CoV-2 diagnostic test results.

### Comparison of serological assays

Samples taken as part of the Co-STARS study [12] which had an accompanying symptom start date available for analysis were initially screened for seropositivity by the EDI assay or by any of the three antigens of the Meso Scale Discovery (MSD) assay. The selected samples underwent testing with 4 serological assays: 1) The Roche Elecsys Anti-SARS-CoV-2 electrochemiluminescence immunoassay (ECLIA) assay detects the nucleocapsid (N) antigen (Roche-N); 2) the Roche Elecsys Anti-SARS-CoV-2 S electrochemiluminescence immunoassay (ECLIA) assay detects the spike (S) antigen (Roche-S); 3) the Abbott Nucleoprotein Chemiluminescent Microparticle Immunoassay (CLMIA) assay detects the nucleocapsid (N) antigen (Abbott-N); 4) The four antigen Meso Scale Discovery (MSD) assay was undertaken at the WHO Pneumococcal Supranational Reference Laboratory at the UCL Institute of Child Health. Only 3 antigens were reported from the MSD assay (the Spike, the Nucleoprotein and the receptor binding domain, RBD) as the baseline test performance of the N-terminal domain (NTD) antibody response was insufficient for further evaluation as previously reported [13]. The Roche-N and Roche-S assays were undertaken by the Laboratory Medicine Service of Swansea Bay University Health Board, Morriston Hospital, Swansea. The Abbott-N assay was undertaken by Public Health Wales Microbiology at Cardiff and Vale University Hospital. All samples were stored and transported between laboratories at −80oC and only removed for aliquoting prior to testing to avoid unnecessary freeze-thaw cycles.

### Statistical Analysis and Modelling

In order to evaluate the relative proportion of seropositive tests in the parallel serological assays over time, a time-to-event analysis was performed using the time from symptom onset and the first negative test for each assay after a first positive test as the event of interest using the R package *survival* [14, 15]. Only tests taken >14 days after symptom onset were considered in the analysis (albeit no tests were performed between 14- and 21-days post symptoms meaning >14 or >21 days post symptom results were identical). A participant was defined as seropositive when at least one of the 4 tests undertaken was seropositive. If the other tests that were run in parallel never became seropositive, the time-to-event was set to the earliest test taken for that individual. If a participant never became seronegative during the follow-up period, a right-censored observation was added at the time of the last serological test. Additionally, the decay rate after 21 days since symptom onset was estimated using a Bayesian generalized mixed model, where time from symptom onset was included as a fixed effect and study participants as a random effect. The decay rate was estimated from the slope of the regression model.

## Results

A total of 950 samples from 329 participants seropositive by any assay after 14 days underwent testing with the Roche-N, Roche-S, the MSD and the Abbott-N assay. The majority of the participants (98%, 321/329) had a positive result by two or more assays.

### Antibody Decay with Time

**Figure 1:**
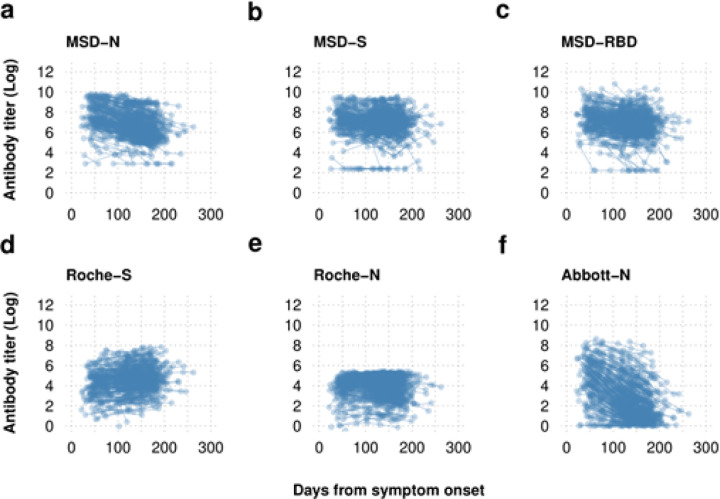
Log transformed serial serological antibody titre data plotted by time from symptom onset Antibody dynamics are dependent on the assay used with the sensitive Roche-S and MSD-S assay demonstrating maintenance of the spike protein antibody while the nucleoprotein antibody is shown to wane with the MSD and Abbott-N assays but to a lesser extent with the Roche-N assay.

Plotting the raw log transformed antibody titres over time since symptom onset (Figure 1) demonstrated that antibody dynamics were dependent on the assay undertaken. The production of spike antibodies was demonstrated to be maintained at high levels up to 200 days when evaluated by the MSD and the quantitative Roche -S assay. All nucleoprotein antibody assays demonstrated decay of the nucleoprotein antibody over time. This was most pronounced in the Abbott-N assay and much less so in the Roche -N assay which demonstrated slow waning of the nucleoprotein antibody.

### Assay Sensitivity with Time Post Symptom Onset

The existing published test performance for all assays undertaken is provided in Table 1. The sensitivity of all assays (at least 14 days from symptom onset) at 50, 100 and 150 days is provided in Table 2. All assays demonstrated a reasonable sensitivity at 50 days following infection. As time passed following infection, the Abbott-N assay rapidly became seronegative, with a median survival time inferred at 175 days (95% CI 168–185 days), whereas the survival probability at 150 days was inferred to be 95% for the Roche-N (95% CI 0.92 – 0.97), and 91% for the MSD-N assay (95% CI 0.87 – 0.94). The Roche-S and MSD-S assays remained seropositive for the duration of the study. The MSD-RBD assay showed some evidence of waning seropositivity over time (90% Survival probability at 150 days, 95% CI 0.88 – 0.94).

A total of 45% (159/329) of the individuals had a negative result using the Abbott-N assay during the course of the study. For the MSD test, 16% (52/329) of participants had a negative test for the N antigen, 11% (36/329) for RBD, and 3% (11/329) for the S antigen. For the Roche platform, 5.5% (18/329) of the individuals had a negative result with the Roche-N assay, while only 4.8% (16/329) of them had a negative result for the S antigen over the course of the study.

### Mathematical Model Fits to Estimate Long-Term Antibody Decay

**Figure 2:**
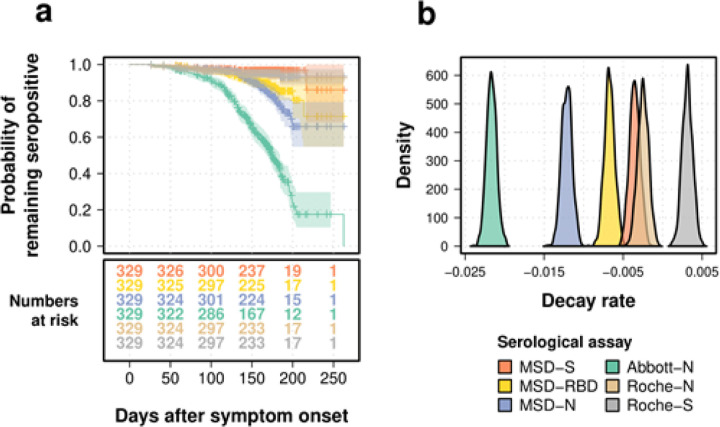
Comparison of seropositivity and antibody dynamics between serological tests. The Roche-S assay target the spike antibody, the Abbott-N and the Roche-N assays target the N-antibody while the MSD assay targets the N-, the S- and the antibody to the Receptor Binding Domain (RBD) of the spike protein in parallel. (a) Kaplan-Meier curve and numbers at risk (the number of participants under follow up with serological tests available for analysis at that time point) for different serological tests. Y-axis represents the probability of remaining seropositive, while the X-axis shows days after symptom onset with numbers of participants under follow up shown in the table below. (b) Inferred posterior density distributions of the decay rate in a generalized linear mixed model.

To estimate the decay rate for each antibody and assay studied, a generalized linear mixed model was fitted to the trajectory of antibody decay after 21 days from symptom onset, where the decay rate was estimated as the slope of the antibody titer through time. Under the most sensitive and quantitative Roche -S assay the spike antibody demonstrated no decay at all and rather a slow rate of increased titres over time from symptom onset (0.0031, 95% CI 0.0018 — 0.0044, Fig. 2b). In accordance with the raw observed data, all nucleoprotein antibodies under the mathematical model decayed (Fig. 2a, Fig. 2b). This was most pronounced in the Abbott-N assay (−0.022, 95% CI −0.023 — −0.02) and least pronounced in the Roche -N assay (−0.0025, 95% CI −0.0039 — −0.0012, Fig. 2b, Table 3).

The Roche-S assay target the spike antibody, the Abbott-N and the Roche-N assays target the N-antibody while the MSD assay targets the N-, the S- and the antibody to the Receptor Binding Domain (RBD) of the spike protein in parallel. (a) Kaplan-Meier curve and numbers at risk (the number of participants under follow up with serological tests available for analysis at that time point) for different serological tests. Y-axis represents the probability of remaining seropositive, while the X-axis shows days after symptom onset with numbers of participants under follow up shown in the table below. (b) Inferred posterior density distributions of the decay rate in a generalized linear mixed model.

**Figure 3:**
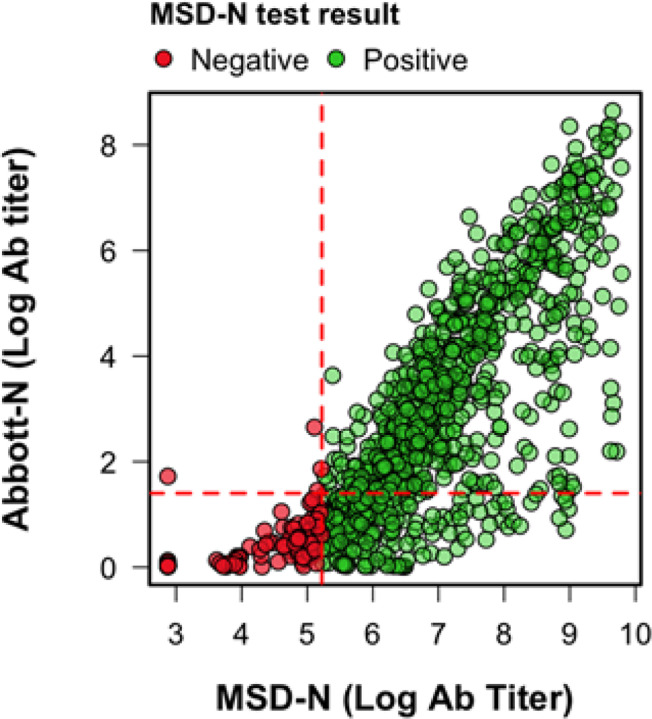
Comparison of antibody titers between the Abbott-N assay and the MSD-N assay. The quantitative results for the MSD-N assay were compared to those of the Abbot-N test for each sample taken. Colours divide the samples depending on whether it was positive (green) or negative (red) for the MSD-N assay. Dotted red lines represent the seropositivity threshold for the Abbott-N assay (horizontal) and the MSD-N test (vertical).

The lower performance of the Abbott-N assay can be explained by a lower detection of titer values as their concentration wanes over time. When compared to the quantitative MSD-N, 26% (222/860) of all positive samples by the MSD-N were negative for the Abbott-N test (Fig. 3). A total of 75% of samples (137/183) positive by the MSD-N with an MSD arbitrary titer value lower than 403 were negative for the Abbott-N assay.

The quantitative results for the MSD-N assay were compared to those of the Abbot-N test for each sample taken. Colours divide the samples depending on whether it was positive (green) or negative (red) for the MSD-N assay. Dotted red lines represent the seropositivity threshold for the Abbott-N assay (horizontal) and the MSD-N test (vertical).

## Discussion

Sensitive measurement of SARS-CoV-2 seropositivity is key to evaluate who has been infected or exposed to SARS-CoV-2, to determine the correlates of protection from future disease, stratify those that need booster vaccination and target the use of anti-SARS-CoV-2 antibodies to those that are seronegative. To our knowledge no other study has evaluated the sensitivity of multiple diagnostic tests in parallel on longitudinally collected serological samples. This study demonstrates that as time elapses after infection, the sensitivity of serological testing varies widely depending on the test used. Although serological tests may be demonstrated to perform well 14–21 days after infection, this initial test performance often diminishes as time passes. In order to evaluate whether or not the population maintains SARS-CoV-2 antibodies it is vital that we utilize serological tests that remain sensitive over time.

Initial published baseline test performance reports concluded that the Abbott-N assay was a high-performance test and a key tool in SARS-CoV-2 surveillance [16]. Our data demonstrate that as time passes following infection the sensitivity of this assay declines rapidly until at <6 months following infection it is no more than 50% sensitive. Our findings support the concerns raised by others regarding the poor performance of some nucleoprotein based assays [19, 20].

In contrast, the Roche assays, particularly the Roche Elecsys Anti-SARS-CoV-2 Spike assay maintained high sensitivity for the 200-day duration of the study. Although there remains no single correlate of sterilizing or protective immunity following SARS-CoV-2 infection or vaccination, it is clear that natural infection and the presence of neutralizing spike antibodies decreases the possibility of re-infection and the severity of disease upon re-exposure to currently circulating strains [21]. Our finding that neutralizing spike antibodies remained at high titres 200 days after infection adds to our previous study on this topic [10] and provides further evidence in support of long-lasting protection against severe disease from currently circulating strains. Fitting mathematical models to the raw data of the Roche spike assay demonstrated that spike antibody titres did not decay but rather increased slightly over the duration of the study. The Roche nucleoprotein assay also maintained sensitivity for the duration of the study with a low rate of decay. Although this assay is semi-quantitative, our findings suggest that this could be used to sensitively identify those that have been vaccinated from those that have been both vaccinated and infected. This has relevance for the diagnosis of long covid, MIS-C and COVID infection post vaccination.

Many studies have evaluated the impact of time on test sensitivity over the first 3 weeks following symptom onset [22–24]. However, we found no other study that had examined the sensitivity of antibody testing on parallel longitudinal samples collected between 1–6 months after infection or exposure. Assays with a higher titer cut-off for detection may perform well in the initial period after infection, but fail to detect seropositivity as antibody levels wane over time. We show that the Abbott-N test failed to detect 75% of samples positive for the MSD-N with a titer value lower than 403, which makes the Abbott-N assay less suitable for seroprevalence studies. Barzin et al [25] used Abbott-N testing alone to determine SARS-CoV-2 seroprevalence in 2,973 asymptomatic out-patients in North Carolina estimating a seroprevalence of 0.8%. Similarly, Wilkins et al [26] used Abbott-N on 6510 healthcare workers up to 150 days after symptom onset and estimated a seroprevalence of 4.8%. Our findings suggest that previously published surveys of SARS-CoV-2 seroprevalence such as these could have significantly underestimated the true prevalence of SARS-CoV-2 humoral immunity.

Memory T-cell interferon gamma release or proliferation assays in response to SARS-CoV-2 antigens provide an alternative means of assessing prior exposure to infection. However, these assays are limited by cross reactive immunity to the seasonal coronaviruses decreasing specificity [27, 28].

Although all serological tests used in the study demonstrated a high initial specificity, one limitation of this study is that only 38% of participants had a confirmatory SARS-CoV-2 PCR result. Our data may therefore be influenced by an unknown proportion of falsely positive serological tests. However, at entry to the study, all seropositive participants had both a screening EDI nucleoprotein assay and an MSD assay performed which limited the chances of a falsely positive result due to a single erroneous test. Not all samples were processed at the same time; the Roche and Abbott-N assays were processed 3 months after the MSD assays. Despite this, we believe that sample storage and freeze-thawing cycles are unlikely to have influenced our findings as the Roche quantitative spike assay was performed last and demonstrated the highest prolonged levels of spike antibody of all tests used.

## Conclusions

In summary, although serological tests may demonstrate high sensitivity 3-weeks after SARS-CoV-2 infection, this is far from the case with some tests 6-months after infection. The Abbott-N assay performed poorly at this time, whereas the Roche and MSD tests maintained a high sensitivity for the 200 days of the study. Tests that perform poorly over time will lead to spurious estimates in population level seroprevalence studies and findings from these studies should be adjusted to account for sensitivity of the test used and the time since infection. Test performance as time passes post infection should be considered before evaluating who is a candidate for booster vaccination or anti-SARS-CoV-2 antibody therapy.

## Figures and Tables

**Table 1:** A summary of the existing published data for the commercially available tests in this comparison. The corresponding antigen target and published sensitivity, specificity, positive predictive value and negative predictive value (if available).

Tests	Manufacturer	Target Antigen	Type	Sens^†^	Spec	# Samples/Patients^*^	Days post symptom onset
Abbott-N [16]	Abbott-N	Nucleoprotein	CLMIA	100.0% (day 17)	99.9%	689/125	≥21
Roche-N [17]	Roche Cobas	Nucleoprotein	ECLIA	99.5%	99.8%	496/102	≥14
Roche-S [18]	Roche Cobas	Spike protein	ECLIA	96.6%	100%	1485^‡^/331	≥15
MSD [13]	Meso Scale Discovery	Nucleoprotein	ECLIA	87.2%	92.8%	196/196	≥21
		Spike protein		97.9%	97.4%	47/47	≥21
		RBD (receptor binding domain)		93.6%	92.3%	47/47	≥21

ECLIA: Electro Chemiluminescent Immunoassay, CLMIA: Chemiluminescent Microparticle Immuno Assay PPV: Positive Predictive Value, NPV: Negative Predictive Value, Sens: Sensitivity, Spec: Specificity, ELISA: Enzyme Linked Immunosorbent Assay

† The highest reported sensitivity

‡ 233 of these samples were tested at ≥15 days post PCR diagnosis

* For Sensitivity Testing

**Table 2:** Sensitivity of compared assays at 50, 100 and 150 days from symptom onset.

	Survival probability (95% CI)		
	50-day	100-day	150-day
**Abbott-N**	0.985 (0.97;0.99)	0.919 (0.89;0.95)	0.655 (0.6;0.71)
**Roche-N**	0.988 (0.98;1.0)	0.963 (0.94;0.98)	0.949 (0.92;0.97)
**Roche-S**	0.991 (0.98;1.0)	0.966 (0.95;0.99)	0.952 (0.93;0.98)
**MSD**			
**-N**	0.988 (0.98;1.0)	0.972 (0.95;0.99)	0.907 (0.87;0.94)
**-S**	0.997 (0.99;1.0)	0.978 (0.96;0.99)	0.968 (0.95;0.99)
**-RBD**	0.994 (0.99;1.0)	0.969 (0.95;0.99)	0.909 (0.88;0.94)

**Table 3:** Decay rate for each serological assay (log arbitrary units per day) estimated in a generalized linear mixed model.

	Mean	95% CI
MSD-N	−0.0121	−0.0134;−0.0107
MSD-RBD	−0.0068	−0.008;−0.0055
MSD-S	−0.0035	−0.0048;−0.0023
Abbott-N	−0.0216	−0.0229;−0.0204
Roche-S	0.0031	0.0018;0.0044
Roche-N	−0.0025	−0.0039;−0.0012

## Abbreviations

SARS-CoV-2: Severe acute respiratory syndrome coronavirus 2
MSD: Meso Scale Discovery
Roche-N: Roche Elecsys-Nucleoprotein assay
Roche-S: Roche Elecsys-Spike assay
Abbott-N: Abbott Nucleoprotein assay
Co-STARs: COVID-19 Staff Testing of Antibody Responses Study
ELISA: Enzyme Linked Immunosorbent Assay
EDI assay: Epitope Diagnostics, Inc.
CLMIA: Chemiluminescent Microparticle Immunoassay
RBD: Receptor binding domain
NTD: N-terminal domain
ECLIA: Electro Chemiluminescent Immunoassay
MIS-C: Multisystem inflammatory syndrome in children
PCR: polymerase chain reaction
CI: confidence interval
